# Sodium acetate increases the productivity of HEK293 cells expressing the ECD-Her1 protein in batch cultures: experimental results and metabolic flux analysis

**DOI:** 10.3389/fbioe.2024.1335898

**Published:** 2024-04-10

**Authors:** Bárbara Ariane Pérez-Fernández, Lisandra Calzadilla, Chiara Enrico Bena, Marco Del Giudice, Carla Bosia, Tammy Boggiano, Roberto Mulet

**Affiliations:** ^1^ Group of Complex Systems and Statistical Physics, Department of Applied Physics, Physics Faculty, University of Havana, Havana, Cuba; ^2^ Center of Molecular Immunology, Havana, Cuba; ^3^ Italian Institute for Genomic Medicine, Candiolo, Italy; ^4^ Department of Applied Science and Technology, Politecnico di Torino, Torino, Italy; ^5^ Group of Complex Systems and Statistical Physics, Department of Theoretical Physics, Physics Faculty, University of Havana, Havana, Cuba

**Keywords:** HEK293 cell line, sodium acetate, heterologous protein, metabolomics, metabolic flux analysis

## Abstract

Human Embryonic Kidney cells (HEK293) are a popular host for recombinant protein expression and production in the biotechnological industry. This has driven within both, the scientific and the engineering communities, the search for strategies to increase their protein productivity. The present work is inserted into this search exploring the impact of adding sodium acetate (NaAc) into a batch culture of HEK293 cells. We monitored, as a function of time, the cell density, many external metabolites, and the supernatant concentration of the heterologous extra-cellular domain ECD-Her1 protein, a protein used to produce a candidate prostate cancer vaccine. We observed that by adding different concentrations of NaAc (0, 4, 6 and 8 mM), the production of ECD-Her1 protein increases consistently with increasing concentration, whereas the carrying capacity of the medium decreases. To understand these results we exploited a combination of experimental and computational techniques. Metabolic Flux Analysis (MFA) was used to infer intracellular metabolic fluxes from the concentration of external metabolites. Moreover, we measured independently the extracellular acidification rate and oxygen consumption rate of the cells. Both approaches support the idea that the addition of NaAc to the culture has a significant impact on the metabolism of the HEK293 cells and that, if properly tuned, enhances the productivity of the heterologous ECD-Her1 protein.

## 1 Introduction

The CHO cell lines, epithelial cells derived from the ovary of the Chinese hamster, have been the predominant mammalian line in the biotechnology industry, particularly for glycoprotein production (Lalonde and Durocher, 2017). However, these cell lines can introduce immunogenic structures that negatively impact the efficacy of some biopharmaceutical products [see, for example, (Seth et al., 2006; Dietmair et al., 2012; Kantardjieff and Zhou, 2014; Phillips, 2014; Ramos et al., 2020)]. As a result, there has been a growing interest within the industry in utilizing human cell lines, such as HEK293 for the production of recombinant proteins.

Human Embryonic Kidney (HEK293) cells are characterized by their high transfectability and their ability to grow in serum-free suspension cultures. These properties make them an attractive host for the expression and production of recombinant proteins (Henry and Durocher, 2011; Dietmair et al., 2012) and an important target in the search of novel strategies to improve protein productivity. These strategies follow two approaches: the modification of genes involved in cellular processes, such as apoptosis (Nishida et al., 2014), cell proliferation (Locard-Paulet et al., 2022), glycosylation (Indellicato and Trinchera, 2021), metabolism (Tarazona and Pourquie, 2020), secretion and protein folding (Abaandou et al., 2021), and the optimization of bio-processes, essentially changing media composition and nutrient feeding protocols (Singh et al., 2017; Galbraith et al., 2018; Pérez-Fernández et al., 2021). The progresses in the field have led to a significant increase in the yield of recombinant proteins, up to a 100-fold improvement, compared to yields obtained two decades ago (Matasci et al., 2008).

However, despite these advances in proteins yield, the industry still demands higher productivity and lower costs. A deeper understanding of the metabolism of these cells and its relationship with the conditions of the culture may open the doors to achieve these goals. For instance, while it is clear that the cell line HEK293 metabolizes glucose inefficiently (Lanks and Li, 1988; Abaandou et al., 2021) and the importance of carbon metabolism is still unclear, most of the metabolic engineering techniques used to increase the productivity are linked to the over-expression of the pyruvate carboxylase (PYC2) gene (Elias et al., 2003; Henry and Durocher, 2011; Vallée et al., 2014; Karengera et al., 2017). The goal in this case is to direct the pyruvate produced from glycolysis towards the formation of oxaloacetate, reducing its availability for lactate production, increasing energy generation in the Krebs cycle (Irani et al., 1999) and decreasing toxicity in the culture (Fernandez-de-Cossío-Díaz et al., 2017). Several studies have revealed the beneficial effects of this genetic modification on the cell density and growth rate. However, there are no consistent results regarding the final protein titer (Karengera et al., 2017). Indeed, the over-expression of the PYC2 gene has been shown to increase (Fogolín et al., 2001), decrease (Wilkens and Gerdtzen, 2015), or leave the cell-specific productivity unchanged (Elias et al., 2003; Henry and Durocher, 2011).

An alternative approach to increase the protein titer is the use of short chain fatty acids (SCFA) in the feeding medium, such as butyrate and valproic acid. For instance (Yang et al., 2014), evaluated the ability of valproic acid to increase monoclonal antibody titers in three different CHO cell lines. In (Wulhfard et al., 2010) valproic acid was used to obtain protein titer increments in CHO cell cultures, suggesting that this molecule affects transgene mRNA levels. On the other hand (Jiang and Sharfstein, 2008), evaluated butyrate over CHO cells producing a monoclonal antibody. They proved the influence of this SCFA on gene accessibility, measuring the heavy and light chain copy numbers by qRT-PCR and correlating these with treated and untreated cells (Grünberg et al., 2003) studied the impact of sodium butyrate on the efficient production of recombinant antibody fragments in HEK293 cells. The main results suggested that this compound can enhance the production of proteins of interest in this cellular system. More recently (Cervera et al., 2015), explored the use of lithium acetate, valproic acid, sodium butyrate, and others as enhancer additives for the production of virus-like particles during transient transfection of HEK293 cells. They found that the use of these additives, which interact with histone deacetylase inhibitors (Waldecker et al., 2008; Wulhfard et al., 2010; Shackley et al., 2020; Saleri et al., 2022), facilitates the entry of DNA complexes into the cells and increases gene expression levels. Histone acetylation is a significant post-translational modification of proteins that promotes a more accessible chromatin structure, allowing the activation of transcriptional processes (Xu et al., 2007). In this study, we explored a similar strategy to increase HEK293 protein titer by adding sodium acetate (NaAc) to the feeding medium of the culture of HEK293 cell line producing the ECD-Her1.

The ability to use acetate as a carbon source is common to numerous microorganisms, from bacteria to eukaryotes. Acetate belongs to SCFA species and it is an important nutrient that supports metabolism, lipogenesis, and acetylation of proteins by acetyl-coenzyme A (Ac-CoA). It has been previously used in mammalian cell culture studies, showing promising results (Chen et al., 2015; Leone et al., 2015; Liu et al., 2018; Qiu et al., 2019; Kutscha and Pflügl, 2020).

On the other hand, the protein ECD-Her1 is one of the four extracellular domains of the epidermal growth factor receptor (EGFR) pivotal in anti-cancer vaccine development León et al. (2009); Duardo et al. (2015); Caballero et al. (2017); Enrico Bena (2019). Its potential as a therapeutic vaccine has been demonstrated in preclinical studies conducted in mice with lung cancer, highlighting its antimetastatic properties and pro-apoptotic response (Ramírez et al., 2008; Ramírez et al., 2006; Báez et al., 2018). Moreover, it has shown safety and immunogenicity in patients with hormonal castration-resistant prostate carcinoma (Caballero et al., 2017). However, since the protein has a high structural complexity, with 11 N-glycosylation sites and a molecular weight of approximately 105 kDa (Ramírez et al., 2006; Duardo et al., 2015) the HEK293 cell line was chosen as the cellular platform for its production. Currently the Center for Molecular Immunology at Havana, has completed the development stage for ECD-Her1 protein (León et al., 2009). These promising results have motivated our efforts to improve the productivity of the extracellular domain of HER1 (ECD-HER1) protein and in particular to explore the influence of adding NaAc in the medium. To do so, we measured macroscopic parameters relevant to the cell culture, such as cell density, glucose consumption and lactate production, and inferred the actual behavior of the internal metabolism of the cells. We expect, that the knowledge gained studying this process could be also useful for the comprehension and improvement of the production processes of other complex proteins.

## 2 Materials and methods

### 2.1 Experimental techniques

The HEK293 cell line utilized in this study was kindly provided by Dr. Belinda Sanchez (Ramírez et al., 2006). The cloning process and transfection strategy were previously described by Ramírez et al. (2006). The cultivation of the cells producing the ECD-Her1 protein was carried out in suspension mode, facilitating sampling from the culture through the homogenization of samples with a pipette before extraction. Moreover, extensive development and safety evaluations, including immunogenicity assessments conducted during phase I clinical trials (Caballero et al., 2017) have been performed on this HEK293 cell line when employed for ECD-Her1 protein production.

Cell culture started adding the inoculum to a homemade protein- and serum-free medium previously loaded into flasks. We employed 75 cm^2^ T-flasks at 20 mL of working volume in a shaking incubator at (37°C ± 1°C) and 5% of CO_2_ atmosphere.

To quantify the effect of NaAc on growth rate, metabolism and heterologous protein expression of the cell populations, we supplemented the culture medium with different concentrations of NaAc (0 mM, 4 mM, 6 mM and 8 mM). For all conditions, cells were seeded at (5 ± 1) × 10^5^ cells/mL and all the experiments were conducted in duplicated.

The cell growth was monitored daily until cell viability was lower than 70% by taking at least two representative samples of the population. An aliquot of the supernatant was stored daily to quantify the concentration of specific metabolites and ECD-Her1 protein production over time.

### 2.2 Analytical support

The analytical techniques and protocols for measuring cell density, specific protein and metabolite concentrations are described below.

• Viable cell density and viability were quantified by optical microscopy technique using trypan blue dye exclusion method in a Neubauer chamber (Marienfeld company, Germany).
• The ECD-Her1 protein concentration was measured by a homemade ELISA sandwich method (reagents from R*&*D Systems (Minneapolis, United States of America)). Briefly, 96-microwell plates were coated with 5 mg/mL anti-ECD-Her1 and incubated overnight at 4°C. Subsequently, samples and controls were added to the plates and incubated at 37°C for 1 hour. An anti-EGFR antibody conjugated with biotin was added to the plate and incubated at 37°C for 1 hour. Streptavidin-peroxidase reagent was then added to the plate and incubated at 37°C for 1 hour. Finally, tetramethylbenzidine substrate was added and after 20 min, the reaction was stopped, and the plates were read using a spectrophotometer at 450 nm. To be sure that the results were associated to the modification in the culture conditions the method was used with validated reference materials as controls, all the samples and controls were run in the plate in triplicate, and the coefficient of variation was kept lower than 15%. Concerning buffers, the plate was first coated with 5 mg/mL of anti-ECD-Her1 [Nimotuzumab, homemade antibody (Rodríguez et al., 2013)], prepared in carbonate buffer 0.1 mM and pH = 9.6. The PBS-tween20 (0.05%) buffer was used to carry out the washing steps, but also to prepare anti-EGFR antibody conjugated with biotin and Streptavidin-peroxidase reagent [both from R*&*D Systems (Minneapolis, United States)].
• Metabolite concentrations were obtained through a Liquid Chromatographic-Mass Spectrometry (LC-MS) analytical method, which offer high resolution ( > 100,000) and accurate mass quantification (routinely <2 ppm) as mentioned in (Mackay et al., 2015a) previously described by Mackay et al. (2015b). We used the same number of replicates used for the cell culture experiments (two replicates per condition). We obtained, Glucose, Lactate and other 70 extracellular metabolites. To corroborate the technique’s precision regarding amino acids and key metabolites, we also used control samples corresponding to whole medium Advanced DMEM/F-12 (Gibco, reference number 12634010) supplemented with glutamine and lactate at specific molar concentrations.

The first step of processing corresponds to the extraction of metabolites from the samples using Acetonitrile, Methanol and MilliQ water (3:5:2 ratio) as the extraction solvent. Later, the measurement in the LC-MS was done by using the configuration composed of Heater Electro Spray Ionization source (HESI), ORBITRAP detector (AGILENT company, CA, United States) and ZIC-pHILIC column (MERCK Millipore company, Germany).

Metabolites were separated using the LC column depending on their retention times. We used an aqueous mobile phase solvent, 20 mM ammonium carbonate, adjusted to pH 9.4 with a 0.1% ammonium hydroxide solution (25%), and an organic mobile phase of 100% acetonitrile. A linear gradient scheme was applied for the separation process at 200 mL/min taking the application for approximately 15 min, followed by an equilibration step. The column was kept in an oven at 45°C and the samples were maintained at 4°C prior to injection into the mass spectrometer. Finally, the metabolites were isolated by their mass/charge (m/z) with mass accuracy below 5 ppm for all metabolites using a Q-Exactive mass spectrometer. All standard reagents used for the quantification of the metabolites involved in the process were obtained from SIGMA ALDRICH (Merck Millipore, Germany).

### 2.3 Extracellular flux analysis: experimental description and protocols

For the extracellular flux analysis (EFA), in order to study glycolysis and mitochondrial function (Tian et al., 2021) we employed a Seahorse XFe96 instrument (Agilent technology, California, United States). All the accessories utilities, reagents, medium and kits are also from Agilent technology. The machine is equipped with 96 well chamber, each one with pH and oxygen sensors. Inside the chamber, the temperature is kept constant at 37°C during the entire measuring process. A detailed description is presented below.

• Medium and reagents

The medium used for the Seahorse experiments was XF-DMEM to pH 7.4 supplemented with 15 mM of XF-Glucose (1.0 M stock solution), 1 mM of XF-Pyruvate (100 mM stock solution) and 2 mM of XF-Glutamine (200 mM stock solution). The Seahorse XF Cell Mito Stress Test Kit and the Seahorse XF Cell glycolytic rate Test Kit were prepared before used, following the manufacturers’recommendations.

• Adaptation process

The adaptation process for both the control and NaAc conditions was carried out gradually, taking into account that the cells originated from the serum-free medium mentioned earlier. To achieve this, cells were incrementally acclimated every 48–72 h by blending the new medium (1) with the existing one (2) in varying volumetric ratios: 25%–75%, 50%–50%, 75%–25%, and finally 100%–0%. Each blend was maintained for three consecutive passages, while closely monitoring cell density and viability throughout the entire process.

• Cell culture treatment

For the evaluation of glycolytic and mitochondrial metabolism, cells were divided in two groups. In the first group, from here on referred to as “control”, cells were grown in complete DMEM + Fetal bovine serum (10%) (GIBCO, United States). In the second group, from here on referred to as “NaAc condition”, cells were grown in the same complete medium of the control supplemented with 8 mM of NaAc. The process of adaptation of both control and NaAc condition was done gradually, considering that cells came from the serum free medium described above. This protocol was carried out 3 months before the experiments in order to make sure that cells belonging to the control and the NaAc condition were realizing different metabolisms.

• Extracellular Flux Analysis protocol

The procedures we followed to measure glycolytic rate and mitochondrial respiration were previously described in (Qing et al., 2021; Gu et al., 2021), respectively. Briefly, 10,000 cells in 80 *μ*L of complete medium were seeded in a sterile Seahorse XF96 cell culture Microplate, 48 h prior to the Seahorse running, with the idea of having 80% of confluency at the moment of the assay. After seeding, the plate was left in the cabin hood for 1 hour and then put at 37°C in a 5%-CO_2_ atmosphere incubator. The day before the assay, the sensory cartridge was hydrated by putting it inside the utility plate with 200 *μ*L Seahorse XF calibrant solution, avoiding bubbles in the process and making sure the sensors were submerged in the calibrant. Then, the sensory cartridge was incubated in a non-CO_2_ incubator at 37°C, up to the assay running. At the same time the Seahorse system was switched on in order to give time to stabilize the instrument to a constant temperature of 37°C. The day of the assay, the XF-DMEM Seahorse medium was supplemented as we described in the previous section, and warmed up to 37°C in a water bath. After that, cells in the microplate were gently washed with 180 *μ*L of warmed medium three times. We then completed the microplate with the same volume of a specific Seahorse medium one last time and incubated in a non-CO_2_ incubator at 37°C for 1 hour. Twenty minutes before loading the cell plate to the machine, we loaded the sensory cartridge plate and wait for the stabilization of the parameters of the Seahorse stabilized. Then, the cell plate was loaded to the machine, and each assay was done as explained below.

• Glycolytic rate assay

The Glycolytic rate test kit was used to carry out the assay. The reagents of the kit, Rotenone (Rot)/Antimycine A (AA), and 2-deoxy-D-glucose (2-DG), were reconstituted in XF-DMEM medium, considering the manufacturer’ protocols and other published researches (Agilent Manual Part Number 103344-400) (Qing et al., 2021).

In the Glycolytic test we used Rot/AA with a stock solution concentration of 50 *μ*M, a volume of assay medium of 540 *μ*L, a concentration in well of 0.5 *μ*M, and a compound injection volume of 20 *μ*L. Further, we used 2-DG with a stock solution concentration of 500 mM, a volume of assay medium of 3,000 *μ*L, a concentration in well of 50 mM, and a compound injection volume of 22 *μ*L.

• Mito stress assay

To carry out the measurement of mitochondrial respiration we used the Mito stress test. The reagents of the kit, oligomycin, carbonyl cyanide-4-(trifluorome-thoxy)phenylhydrazone (FCCP) and rotenone (Rot)/antimycin A (AA), were reconstituted in XF-DMEM medium, considering the manufacturer’ protocols and other published researches [Manual Part Number 103016-400 (Gu et al., 2021)].

In the Mito Stress test, we used oligomycin with a stock solution concentration of 100 *μ*M, a volume of assay medium of 630 *μ*L, a concentration in well of 1.5 *μ*M, and a compound injection volume of 20 *μ*L. Further, we used FCCP, with a stock solution concentration of 100 mM, a volume of assay medium of 720 *μ*L, a concentration in well of 1.0 mM, and a compound injection volume of 22 *μ*L. And we used Rot/AA with a stock solution concentration of 50 *μ*M, a volume of assay medium of 540 *μ*L, a concentration in well of 0.5 *μ*M, and a compound injection volume of 25 *μ*L.

• Software

To run and analyze both Glycolytic rate and Mito stress protocols, we use the software Wave V2.6.3.5 from Agilent technologies.

### 2.4 Metabolic flux analysis

To carry out a metabolic analysis, we used the specific uptake of the metabolites involved. This was computed from the concentrations of external metabolites, the cell density, and the effective cell growth rate. For this, we use the fundamental dynamical equations describing the system:

$$\frac{dX}{dt}=\mu X$$ (1)

$$\frac{ds_{i}}{dt}=-u_{i}X$$ (2)

where X denotes the cell density (units: gDW/L), *μ* the specific cell growth rate (units: 1/h). The term *u*_*i*_ denotes the specific uptake of metabolite i (units: mmol/gDW/h), and *s*_*i*_ is the concentration of metabolite *i* in the culture (units: mM).

Solving Eqs 1, 2 we obtained the specific uptake of experimentally measured metabolites:

$$u_{i}=-\mu \frac{ds_{i}/dt}{dX/dt}$$ (3)

Then, to quantify the intracellular metabolic fluxes, we use Metabolic Flux Analysis, (MFA) (Antoniewicz, 2021; Antoniewicz, 2015). In short, the intracellular fluxes (**v**) are limited by the stoichiometric matrix (**S**):

S⋅v=0 (4)

To determine intracellular fluxes (*v*) from measured external rates (*r*_*m*_), the following least squares problem is solved:

$$\begin{array}{ll} & \text{Min}\;\underset{i}{\sum }{\left(r_{i}-r_{m,i}\right)}^{2}\;\text{for each metabolite }i\\ & \;\text{s.t. }r_{i}+\underset{j}{\sum }S_{i,j}v_{j}=q_{p}p_{i}+y_{i}z \end{array}$$

where *r*_*i*_ are the specific uptakes of experimentally measured metabolites, *p*_*i*_ is a vector with the coefficient of each metabolite to produce 1 gram of protein, q_*p*_ is the specific production rate and *y*_*i*_ are the coefficients of each metabolite to produce one unit of biomass.

We delimited the cell growth rate and specific productivity to experimental values as follows:

$$\begin{array}{ll} & 0.99\mu_{\left(exp\right)}\leq z\leq 1.01\mu_{\left(exp\right)}\\ & 0.99q_{p\left(exp\right)}\leq q_{p}\leq 1.01q_{p\left(exp\right)} \end{array}$$

where *μ*_(*exp*)_ and *q*_*p*(*exp*)_ are the experimental values for the cell growth rate and specific productivity.

Also, we split reversible reaction fluxes into negative and positive parts, $r_{k}=r_{k}^{+}-r_{k}^{-}$ , with $r_{k}^{\pm }\geq 0$ , and quantified the total cost of a flux distribution in the simplest (approximate) linear form (Fernandez-de-Cossío-Díaz et al., 2017):

$$\alpha =\underset{k}{\sum }\alpha_{k}^{+}r_{k}^{+}+\alpha_{k}^{-}r_{k}^{-}\leq C\;\text{Enzymatic costs}$$ (5)

where $\alpha_{k}^{+}$ and $\alpha_{k}^{-}$ are constant flux costs and *k* indicates the reaction. The limited budget of the cell to support enzymatic reactions is modeled as a constraint *α* ≤ *C*, where *C* is a constant maximum cost, according to (Shlomi et al., 2011).

It is worth to mention that our data lacks the 13C labeling that have been used in other experiments. However, the use of isotopic labeling patterns in mammalian cell metabolism, particularly when complex nutritional media is required, introduces additional complexity compared to microbial cultures in minimal media (Orman et al., 2011). This makes the use of the technique and the interpretation of the results particularly challenging. In short, as the number of isotopes and reactions in the metabolic model of the cell increases, the mathematical equations used to identify labeling patterns become significantly more complex. On the other hand, MFA is a much simpler technique. It is based solely on the stoichiometry of metabolic reactions and the measured extracellular metabolite concentrations is computationally efficient and can be applied to a wide range of cellular systems without the need for extensive experimental setup or isotopic labeling. Moreover, it has been also extensively used by the scientific community (Altamirano et al., 2001; Chan et al., 2003; Banta et al., 2005; Banta et al., 2007; Niklas et al., 2009; Quek et al., 2010; Ahn and Antoniewicz, 2011; Martínez et al., 2015).

### 2.5 Metabolic network

The metabolic network used contains 345 metabolites and 365 reactions (Martínez-Monge et al., 2019). This network is an adaptation of a network widely used in the literature for HEK293 cells (Kontoravdi et al., 2007; Wolfe, 2008; Henry and Durocher, 2010; Henry and Durocher, 2011; Henry et al., 2011; Niklas et al., 2011; Kutscha and Pflügl, 2020; Abaandou et al., 2021).

The original network was obtained from a metabolic model reduced following the protocol described by Quek (Quek et al., 2014) for adaptation of the *Homo sapiens* Recon2.0 (Thiele et al., 2013) model for HEK293 cells. Thus, in the first step, Quek revised Recon2 correcting minor bugs, and the resulting model was available from the Biomodels database [MODEL1504080000 (Li et al., 2010)]. In the second step, Quek adapted the resulting model to the specific context of the HEK293 cell culture. In our case, we also added 11 reactions and 10 metabolites that connected with the consumption of acetate and the production of acetyl-CoA and ATP (see Supplementary Material).

## 3 Results

### 3.1 ECD-Her1 protein production increases independently of exponential and stationary phase of cell growth

**FIGURE 1:**
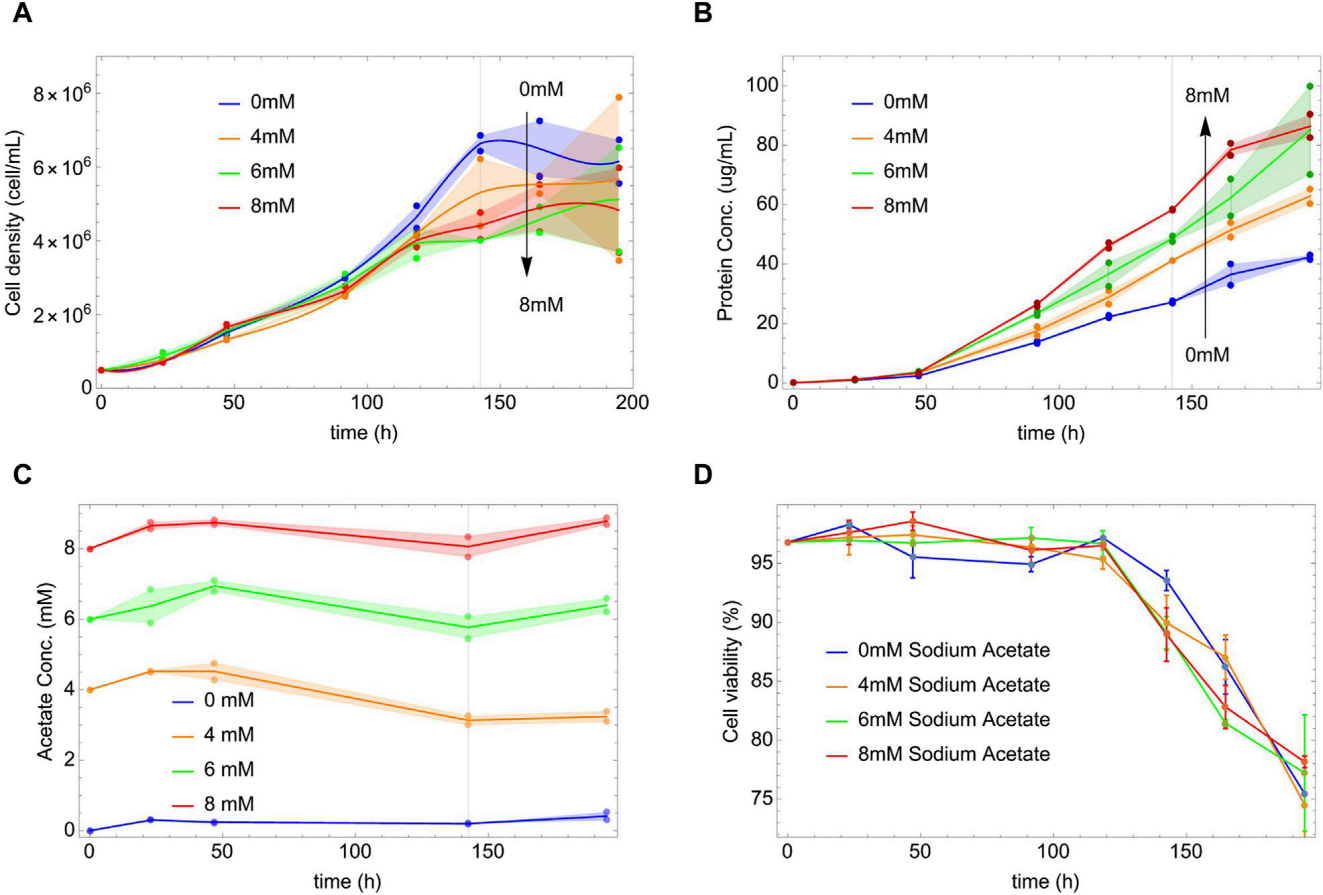
Cell density, protein and acetate concentrations versus time at different initial concentrations of NaAc (0, 4, 6, and 8 mM). Dots represent the experimental data. Shaded areas show the difference between the replicas and the lines are a guide through the mean values. Vertical lines indicate the end of the exponential phases, defined by the stationarity of the cell density in panel **(A)**. Panel **(A)** Each line is an interpolation using the mean of cell density measured at each time. Panel **(B)** Lines join the mean of the protein concentration values measured at each time. Panel **(C)** Acetate concentration versus time at different initial concentrations of NaAc (0 mM, 4 mM, 6 mM and 8 mM). Dots represent the experimental data and lines join the mean of the protein concentration values measured at each time. Shaded areas show the difference between the replicas. Panel **(D)** Cell viability as a function of time for different concentrations of NaAc.

In Figure 1A, we represent the evolution of the density of cells *X* as a function of time for each concentration of NaAc. In all the cases the evolution of *X* shows first, an exponential phase, and later a stationary phase where the cell density is essentially constant. The exponential phase reaches the 144 h and then the stationary phase starts. (See also the data in log scale in the Supplementary Material where we show a liner fit to the exponential phase).

Initially and for most of the exponential phase, the cell density (Figure 1A) appears insensitive to the presence of NaAc; however, after 120 h, the curves reach their nutritional deep and start to differentiate. The higher the dose of NaAc, the lower the nutritional deep and cell density X. A similar phenomenon was observed by McMurray-Beaulieu et al. (2009) that studied the role of sodium butyrate in a culture of CHO cells. The authors, considering the decrease in the viability response, that is also observed with other SCFA (Backliwal et al., 2008; Jiang and Sharfstein, 2008; Salimi et al., 2017), suggested a possible cytotoxic effect (McMurray-Beaulieu et al., 2009) of the sodium butyrate. However, in our case, the presence of NaAc does not affect the culture viability if compared with the control condition (see Figure 1D). Therefore, we think that our experimental findings are the results of more complex intracellular changes, metabolic and/or regulatory. Notice that in the Supplementary Material we show similar, although preliminary, results comparing the effects of larger concentrations of NaAc.

Given the evolution of *X*, it is surprising that the presence of NaAc increases by a factor of two the rate of protein production (see Figure 1B). This occurs, already early in time within the exponential phase, and without any sign of saturation within the evaluated time scale. It is independent of the fact that, after 144 h, the cell density was already stationary or depleted (see Figure 1A). As far as we know, there are not previous reports in HEK293 cells modified to produce a heterologous protein.

However, notice that while other SCFAs, have been used to increase heterologous proteins production (McMurray-Beaulieu et al., 2009; Camire et al., 2017) this is not the case of NaAc where the results are more contradictories. In general, much effort has been put to support two possible mechanisms behind the role of SCFA’s in the cell productivity. Either the metabolism of acetyl-CoA in the ATP cycle (mitochondria), as cofactor for the histone acetyltransferases (HATs) catalysis (Han et al., 2018; Liu et al., 2018; Bose et al., 2019); or directly in the nucleus as inhibitors of histone deacetylase (HDAC) (Thomas and Denu, 2021). Nevertheless, most of the time these studies neglect the NaAc from these mechanisms (Evertts et al., 2013; Han et al., 2018; Thomas and Denu, 2021), unless the cells have been exposed to genetic modifications (Zhao et al., 2016) or hypoxic conditions (Kamphorst et al., 2014).

Figure 1C shows the external concentrations of acetate in the culture as a function of time. As can be seen, it is essentially constant throughout the entire process. At later times, in the exponential phase, we can observe a slight decrease in acetate concentration for 4, 6 and 8 mM NaAc. (Kamphorst et al., 2014) used acetate in lower concentrations (from 50 to 500 *μ*M) and found that under hypoxic conditions, cells used the exogenous acetate as the main source to generate Acetyl-CoA, even in the presence of 25 mM of glucose and 4 mM of glutamine in the culture medium. Also (Zhao et al., 2016), used in their study 13C-labeled acetate at a concentration of 100 *μ*M to find its contribution to the production of Acetyl-CoA in the absence of ATP-citrate lyase (ACLY), concluding that physiological levels are sufficient to generate large quantities of Acetyl-CoA and to maintain the cell viability. In addition to these results, we will show below that even small quantities of NaAc could be enough to drive the protein production. Finally, the viability of the culture is represented in Figure 1D; as can be seen it is independent of the concentration of NaAc.

### 3.2 Sodium acetate dose-dependent response of metabolite uptake and production rates

To understand the metabolic effect of sodium acetate on HEK293 cell culture and protein production, we measured the concentrations of various external metabolites listed in Data availability statement.

**FIGURE 2:**
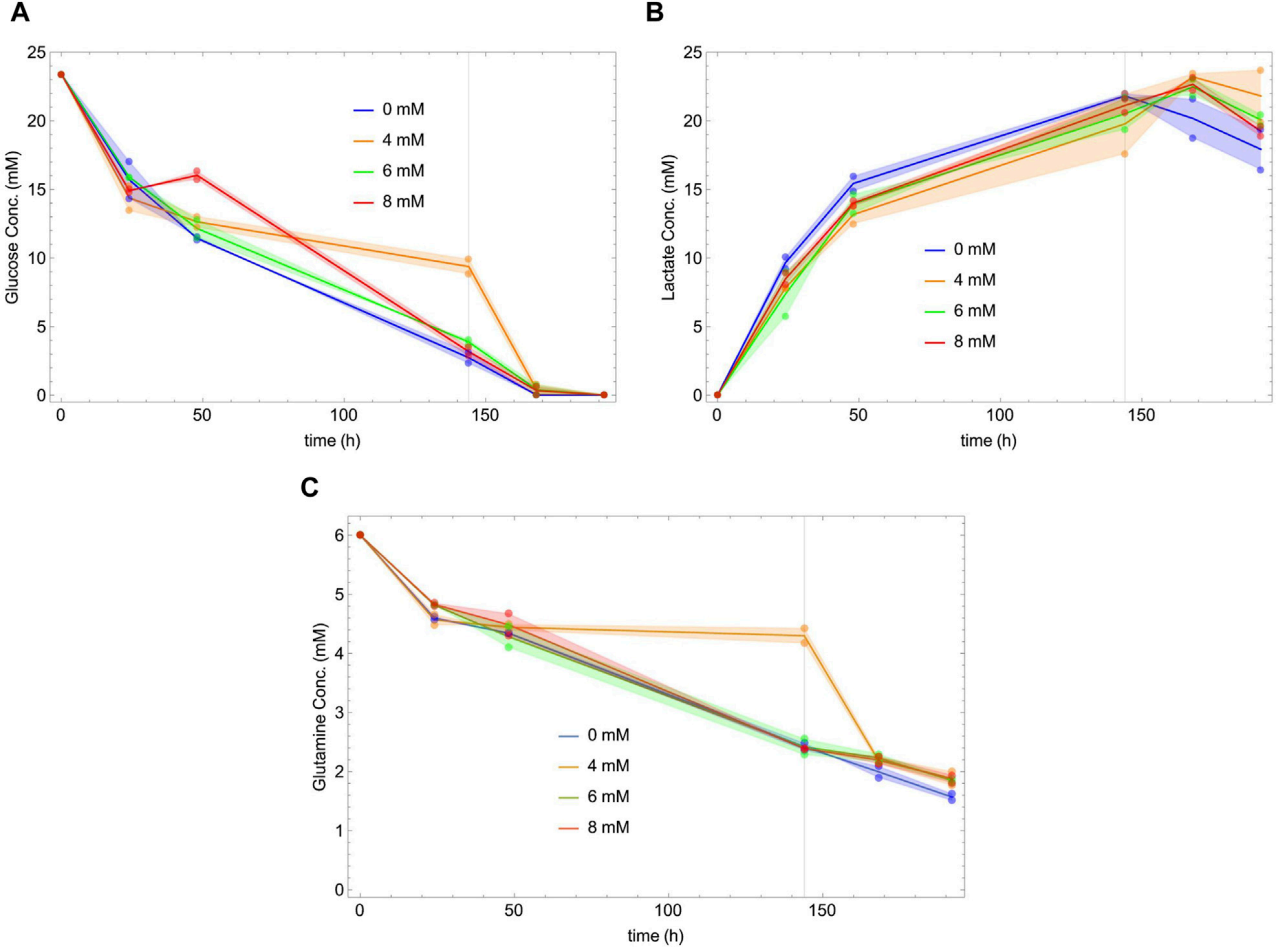
Glucose **(A)**, lactate **(B)** and glutamine **(C)** concentrations versus time at different initial concentrations of NaAc (0 mM, 4 mM, 6 mM and 8 mM). Dots represent the experimental data and lines join the mean of the values measured at each time. Shaded areas show the difference between the replicas. Vertical lines indicate the end of the exponential phases, defined by the stationarity of the cell density in Figure 1A.

Figure 2 shows the behavior as a function of time of glucose (panel A), lactate (panel B) and glutamine (panel C) for different concentrations of NaAc. During the exponential phase, cells consume glucose and glutamine, and secrete lactate. In the second, stationary phase, the cells continue to consume glutamine, and begin to consume lactate. This metabolic change towards the consumption of lactate is well known (Passarella et al., 2008; Luo et al., 2012; Mulukutla et al., 2012; Martínez et al., 2013; Liste-Calleja et al., 2015; Passarella and Schurr, 2018). Notice that there is no evident dependency of the glucose, glutamine and lactate concentrations on the presence of NaAc.

**FIGURE 3:**
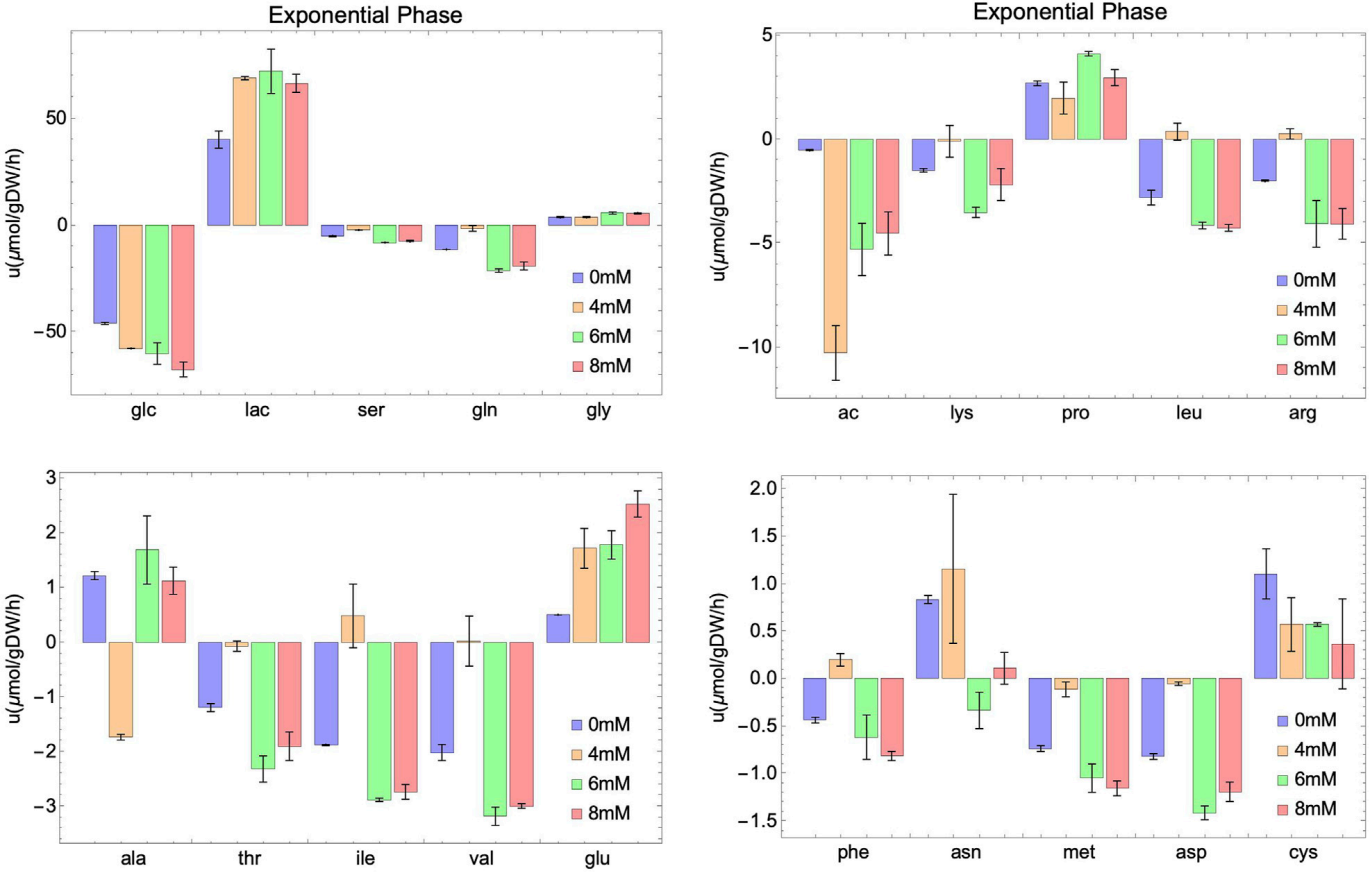
Experimental rates of consumption (negative value) or secretion (positive value) of external metabolites at different initial concentrations of NaAc (0 mM, 4 mM, 6 mM and 8 mM) in the exponential phase. Intervals represent the two replicas for each condition and columns show the mean value of both replicas.

**FIGURE 4:**
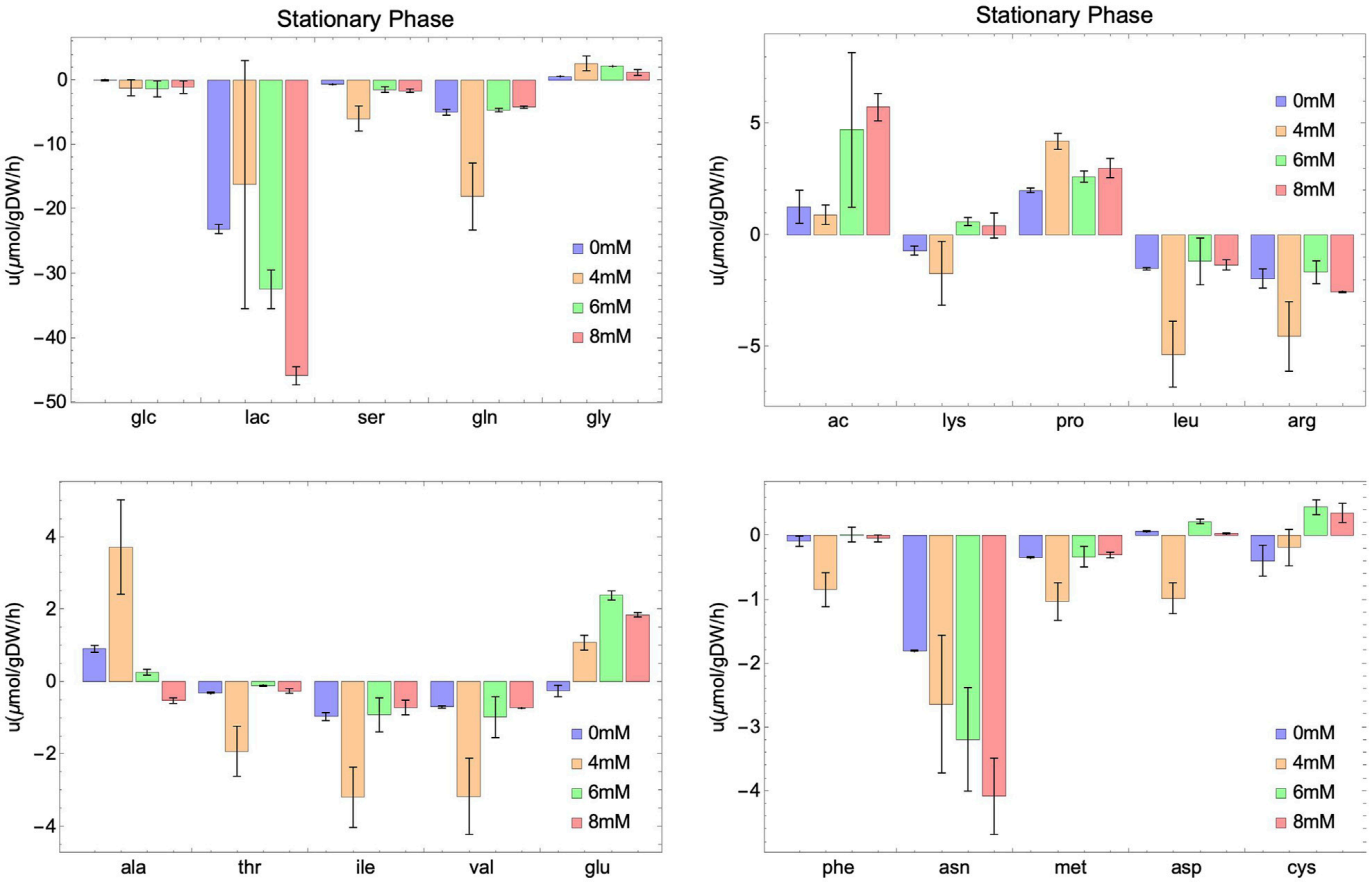
Experimental rates of consumption (negative value) or secretion (positive value) of external metabolites at different initial concentrations of NaAc (0 mM, 4 mM, 6 mM and 8 mM) in the stationary phase. Intervals represent the two replicas for each condition and columns show the mean value of both replicas.

While glucose and lactate concentrations appear to be essentially independent of NaAc, this is not the case of their specific uptakes. The uptake and production rates are represented, together with those of several measured metabolites, in Figures 3, 4. Our data suggest that, regardless of the growth phase of the culture, there is a clear NaAc dose-dependent response of the uptake or production rates of glucose and lactate. Similar results were obtained by (McMurray-Beaulieu et al., 2009) evaluating the effect of sodium butyrate (NaBut) supplementing CHO cultures in specific moments of the growing curve. They measured the concentration of glucose and lactate and, although they are apparently independent of the external concentration of sodium butyrate, their uptake and production rates change.

In the case of glutamine, variations in the consumption rates are noted across different NaAc concentrations compared to the control (Figures 3, 4). However, these variations are smaller than those observed with glucose and lactate. For 4 mM in particular, the behavior seems different, which is consistent with the results obtained for other metabolites.

Several metabolites follow similar patterns, for example, lactate (lac) and asparagine (asn) are first produced (in the exponential phase) and then consumed (in the stationary phase), suggesting that they can be used by cell culture as a potential energy source once the main metabolite in the medium (glucose) is depleted. Notice that a different behavior is observed in acetate. It is consumed in the exponential phase, and secreted in the stationary phase. Others, like serine (ser), methionine (met) and glutamine (gln) are consumed through the whole process, and still some are secreted independently on the growth phase, like glycine (gly), proline (pro) and alanine (ala). A similar behavior for these metabolites is observed in a study carried out by Carinhas et al. (2013). They studied the impact of sodium butyrate on IgG4 protein production in CHO cells using nuclear magnetic resonance and MFA. Their findings revealed an increase in glucose consumption and lactate secretion during the exponential phase upon the addition of NaBut. Moreover, during the stationary phase, there was an elevation in lactate and asparagine consumption. In general, and unfortunately, the number of works studying SCFA and in particular acetate including an extensive metabolic characterization of the culture is limited. Those that exist focus on the production of these metabolites (Millerioux et al., 2012; Liu et al., 2018) instead of the consumption from exogenous sources (Lakhter et al., 2016; Zhao et al., 2016). This work fills also a gap in this direction.

In summary, our results points to an enhancement of the basic response (the one at 0 mM) of the external metabolites during the evolution of the culture, due to the presence of NaAc. As we will show below this reflects directly into the internal metabolism of the cell.

### 3.3 Understanding the internal metabolism: metabolic flux analysis

To understand how differences in specific exchange rates translate into the internal metabolism of the cell, we present here the results of Metabolic Flux Analysis (MFA) (Antoniewicz, 2021; Antoniewicz, 2015) using the HEK293 network (see (Quek et al., 2014) and the Supplementary Material). We concentrated our efforts on extracting the differences between the exponential and stationary phases in cultures supplemented with 0 mM and 8 mM NaAc. The results for 4 and 6 mM are presented in the Supplementary Material.

**FIGURE 5:**
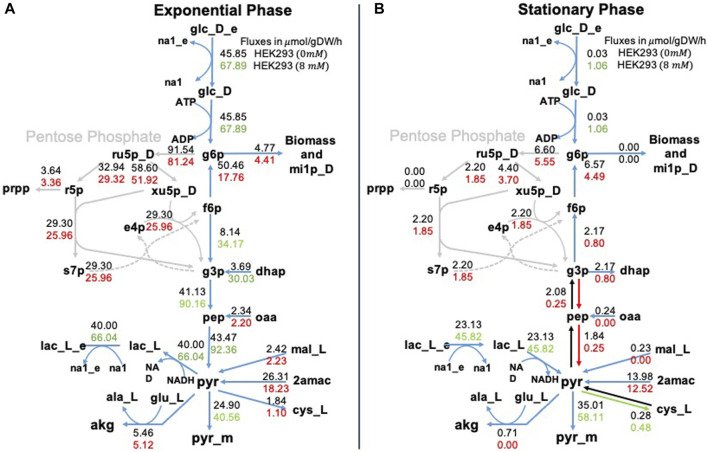
Metabolic fluxes in glycolysis. Representation of the glycolysis pathway in the exponential phase panel **(A)** and in the stationary phase panel **(B)**. We represent two values for each metabolic reaction shown, the top value is the flux of the control (0 mM of NaAc) and the bottom value is the flux at 8 mM of NaAc. We used red to represent fluxes that were lower than the control value, green to represent fluxes that were higher, and black to represent fluxes that were equal. The subscript _e corresponds to extracellular metabolites, _m to mitochondrial metabolites, and the rest of the metabolites are found in the cytosol.

It is convenient to show the results of MFA through metabolic maps that represent the internal metabolic fluxes in the cell. Figure 5 shows the glycolysis pathway in the exponential and stationary phases. Notice that the exchange fluxes are consistent with the experimental results presented above. In the exponential phase, glucose is consumed and lactate is produced, while in the stationary phase, when glucose is depleted, lactate is consumed by the cells. This phenomenology is reinforced in the presence of NaAc. This is a different way to represent the experimental results described in the previous section. However, MFA gives information also about the internal pathways within the cells. For example, in Figure 5A, we can see that during the exponential phase the consumed lactate plays a fundamental role in the production of pyruvate, supporting one of the existing hypotheses in the literature: lactate is converted to pyruvate in the cytosol, and subsequently transported to the mitochondria Mulukutla et al. (2012); Martínez et al. (2013); O’Brien et al. (2020). On the contrary, in Figure 5B, during the stationary phase, the flux of mitochondrial pyruvate to Acetyl-CoA increases in the presence of NaAc. In short, looking into both panels (A and B) of Figure 5 we can conclude that regardless of the phase, when NaAc is present, the values of the fluxes associated with the pentose phosphate pathway decrease.

**FIGURE 6:**
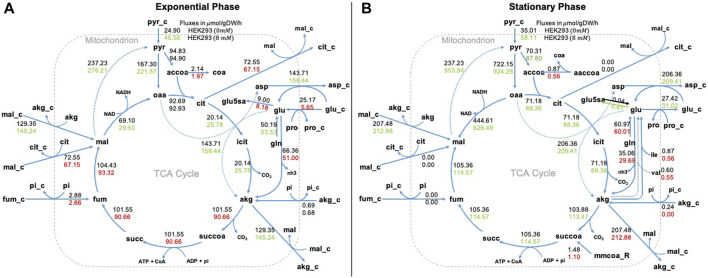
Metabolic fluxes in TCA cycle. Representation of the Krebs or tricarboxylic acid cycle (TCA) in the exponential phase panel **(A)** and in the stationary phase panel **(B)**. We represent two values for each metabolic reaction shown, the top value is the flux of the control (0 mM of NaAc) and the bottom value is the flux at 8 mM of NaAc. We used red to represent fluxes that were lower than the control value, green to represent fluxes that were higher, and black to represent fluxes that were equal. _e corresponds to extracellular metabolites, _c to cytosolic and the rest to mitochondrial.

In Figure 6 we show our results for the Krebs or tricarboxylic acid cycle (TCA). In this pathway the role of NaAc is more evident in the stationary phase. In particular we want to highlight the increase in malate (**mal**_**m**), citrate (**cit**_**m**), isocitrate (**icit**_**m**) and alpha-ketoglutarate (**akg**_**m**) when NaAc is present. This corresponds with the results presented by (Wang et al., 2022) with NaBut, but also with one of the metabolic fates of acetate advocated in the literature (Schug et al., 2016): when glucose oxidation is compromised under hypoxic or low glucose conditions, acetate can be used to generate Acetyl-CoA for energy production through the TCA cycle. In short, MFA confirms that the presence of NaAc in the medium has an impact in the internal metabolism of the cell.

### 3.4 Experimental validation of MFA prediction on internal cell metabolism

Agilent Seahorse extracellular flux technology is a powerful tool that allows real-time measurement of cellular bioenergetics. It is especially useful for monitoring the metabolic activity of cells, including the rate of glycolysis and mitochondrial respiration (Divakaruni et al., 2014). To validate our theoretical predictions, we employed this technique for two different growth conditions of HEK293 cells, both in exponential phase. In one of these conditions, cells have grown without acetate; while in the other, cells adapted in the presence of 8 mM of NaAc.

**FIGURE 7:**
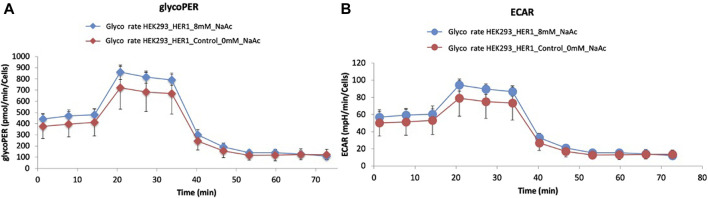
Representative graph of a typical Glycolytic Proton Efflux Rate (glycoPER) **(A)** and Extracellular Acidification Rate (ECAR) **(B)** tests performed with a Seahorse XF96 Flux Analyzer. 25,000 HEK293 cells were seeded per well in 96-well plates and proton efflux was measured for the control (0 mM, red) and for cells adapted to 8 mM of NaAc (blue).

During glycolysis, protons are produced and exported from the cell, leading to the acidification of the extracellular environment (Becker and Deitmer, 2021; Russell et al., 2022). Glycolytic Proton Efflux Rate (glycoPER) is a measure of the rate at which these protons are exported and provides insights about the rate of glycolysis and the metabolic state of the cell. Figure 7A shows the glycoPER rate for HEK293 cells cultured without NaAc (in red) and adapted to 8 mM NaAc (in blue). Despite the overlapping error bars, the trend of the two average values supports the results of MFA (Figure 5): the presence of NaAc increases the rate of glycolysis.

Extracellular acidification rate (ECAR) is another parameter measured with Agilent Seahorse extracellular flux technology. Essentially, ECAR reflects the rate at which cells produce and export lactate and other acidic byproducts of glycolysis to the extracellular environment. Figure 7B illustrates that the average ECAR rate was slightly higher in the presence of acetate. This is also consistent with the increased efflux values for lactate predicted by MFA after the addition of NaAc (see Figure 5A).

**FIGURE 8:**
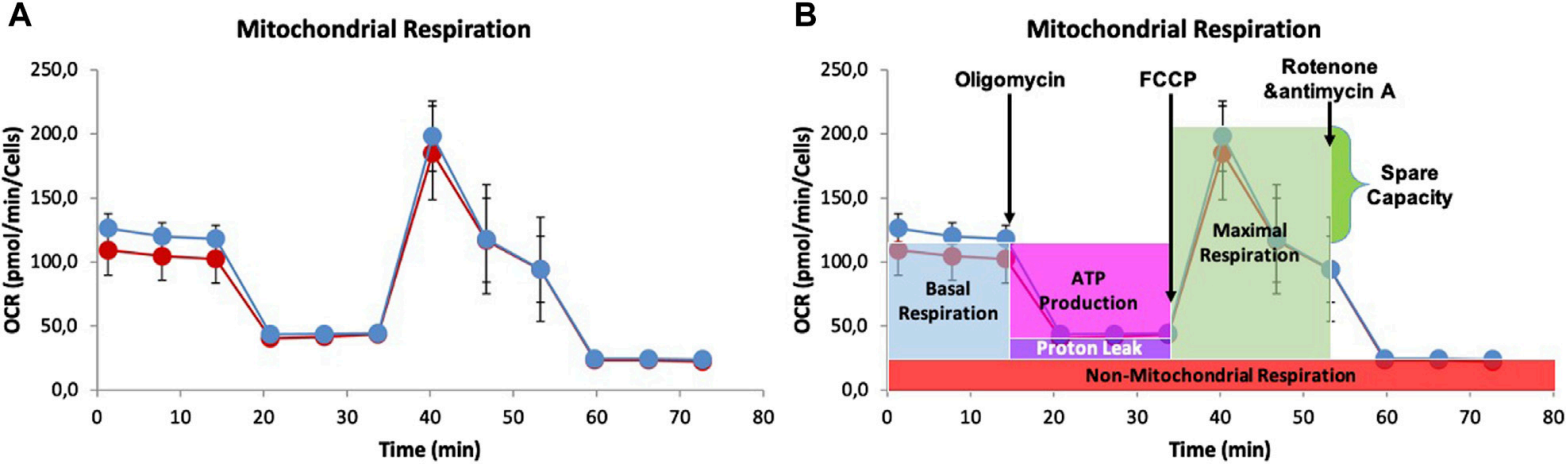
**(A)** Graph of Oxygen Consumption Rate (OCR) measurement of Mito test in living cells HEK293, utilizing the Seahorse XF96 Extracellular Flux Analyzer. 25,000 HEK293 cells were seeded per well in 96-well plates and proton efflux was measured for the control (0 mM, red) and for cells adapted to 8 mM NaAc (blue). **(B)** Representative Seahorse Cell Mito Stress Test assay that shows the fundamental parameters of mithocondrial function: basal respiration, ATP turnover, proton leak, maximal respiration and spare respiratory capacity.

To understand mitochondrial function and identify factors that trigger the switch from healthy oxidative phosphorylation to aerobic glycolysis, we also measured oxygen consumption rate (OCR) (Yépez et al., 2018; Campioni et al., 2022). The results presented in Figure 8 show that there were no significant differences between 0 and 8 mM NaAc, except for the basal respiration during the first 15 min. Since basal respiration can be considered as a threshold below which the cells cannot maintain the oxidative phosphorylation to meet energy demands, Figure 8 suggests that adding NaAc enhances the cells’ ability to meet this energy demand. Notice however, that after these first few minutes, there are hardly any differences between the 0 and 8 mM NaAc groups. This suggests that on larger times the effect of NaAc in mitochondrial respiration is negligible.

**FIGURE 9:**
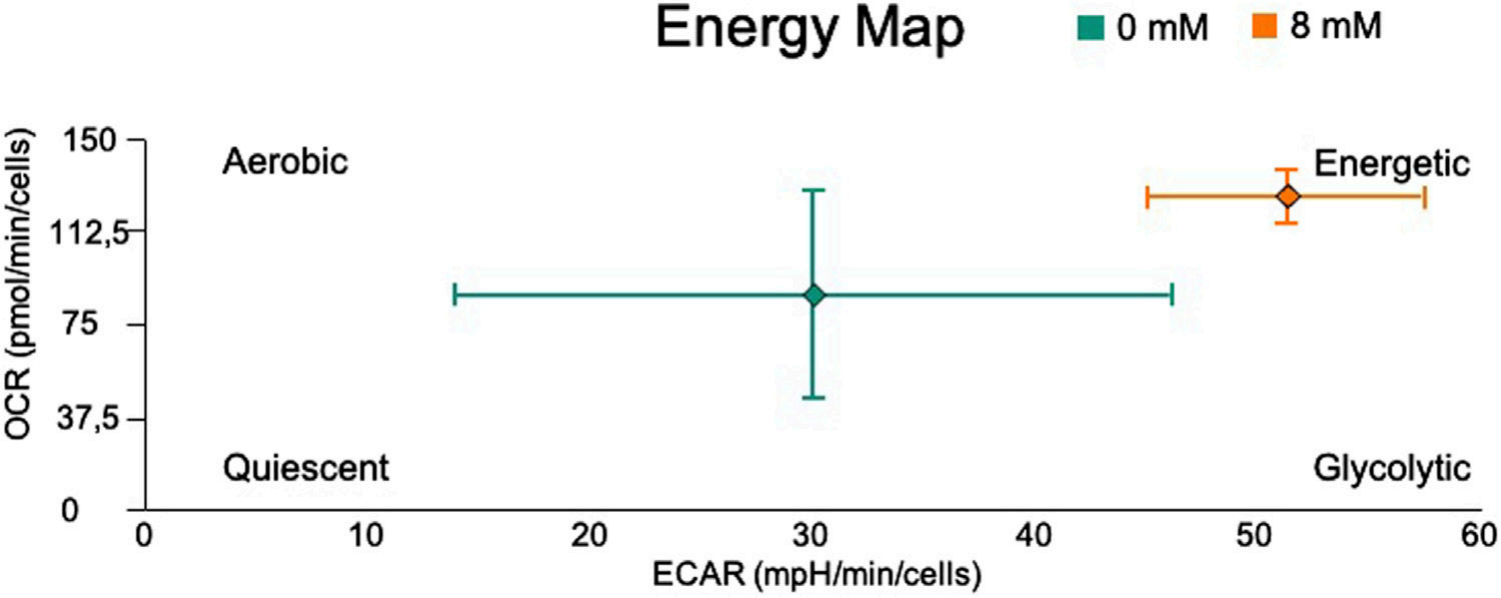
Energy Map of basal OCR vs. basal ECAR in living cells HEK293, utilizing the Seahorse XF96 Extracellular Flux Analyzer. The control (0 mM) is represented with green and cells adapted to 8 mM NaAc with orange.

Summarizing, Figure 9 shows the ECAR and OCR fluxes under basal respiration, and provides a quick overview of the metabolic state of the system (Campioni et al., 2022) with NaAc (in orange) and without NaAc (in green). When NaAc is added, cells increase the glycolytic and respiratory fluxes, indicating a state where the production of ATP is larger than in the control condition at 0 mM NaAc.

### 3.5 On the possible roles of sodium acetate

Based on the results obtained above, we wanted to understand if the small consumption of acetate may promote a change in the energetic metabolism large enough to impact the heterologous protein production. To answer this question, we calculated the energy contribution of acetate in terms of ATP and compared it with the energy required for the formation of the ECD-Her1 specific protein. In short (see Supplementary Material for more details), we estimated this energy requirement as: 8.94 × 10^−3^ mmol of ATP/gDW/h per mole of ECD-Her1. Moreover, the mean difference of acetate concentration (Δ*S*_*ac*_) in our culture (see Figure 1C) was approximately 1.08 mM, indicating a consumption rate of acetate of *u*_*ac*_ ≈ 0.0105 mmol/gDW/h, therefore we estimate the mean quantity of ATP produced by the acetate uptake as, 0.126 mmol of ATP/gDW/h. In other words, the ATP production from the acetate uptake is 14-fold the protein’ ATP requirement. These results indicate that although apparently small, the consumption of acetate might provide the energy necessary for the observed increase in protein production.

Another point of view suggests that sodium acetate is a possible contributor of Acetyl-CoA (Schug et al., 2016), which can be a bioenergetic substrate, a lipogenic precursor and has a significant influence on protein acetylation (Schug et al., 2016). This is a process closely related to histone acetylation and deacetylation, two epigenetic mechanisms that interact in the regulation of gene expression. It involves the transfer of an acetyl group to the terminal amine of the lysine side chains by lysine acetyltransferase (KAT). In cases of hypoxia or starvation, when acetyl-CoA concentrations are low, histone deacetylation occurs, and lysine deacetylases (KDACs) catalyze the hydrolysis of the amide bond to release acetate (McBrian et al., 2013), which is used to produce acetyl-CoA and repeat the acetylation process. Therefore, another explanation for this increase in protein production is that acetate supplementation increases acetyl-CoA concentrations, preventing histone deacetylation, and allowing a more relaxed chromatin structure and the subsequent transcription process in the cells.

## 4 Conclusion

In this study, we explored the role of sodium acetate in the metabolism of HEK293 cells grown in batch. Our results indicate that during the exponential phase, the cell density of the culture does not depend on the presence of sodium acetate, and the nutritional deep decreases when the concentration of NaAc increases. However, cells start to produce protein ECD-Her1 at the beginning of the exponential phase throughout the whole duration of the culture and its productivity increases in the presence of NaAc. We demonstrated experimentally that, while growing exponentially, cells consume glucose and secrete lactate; and then, when glucose is completely depleted, cells start to consume lactate. This phenomenon was also evident in the presence of NaAc. Moreover, we inferred the metabolic flux distribution inside the cell through MFA and the results indicate the presence of the well-known Warburg effect (Vander Heiden et al., 2009; Vazquez et al., 2010; DeBerardinis and Chandel, 2020): the cells increase the uptake of glucose and production of lactate to fuel the metabolic demands despite the presence of oxygen. This effect was more noticeable when NaAc was added. Furthermore, the presence of NaAc increases the fluxes associated with the TCA cycle suggesting a connection with the protein production. To double check the maps that emerged from the MFA, we employed an extracellular flux analyzer (Seahorse XFe96) and monitor the metabolic activity of cells with glycolytic proton efflux rate, and the extracellular acidification and oxygen consumption rates. Again, our results suggest that in presence of NaAc, the cells show an increase in glycolytic and respiratory fluxes, indicating a more energetic state than that of the control sample at 0 mM NaAc. Although we cannot be conclusive about the actual mechanism triggered by NaAc enhancing the protein productivity, our results suggest that, both the direct use of NaAc as a direct source of energy and histone acetylation may be involved. In summary we recommend to use approximately 8 mM NaAc to maximize the production of the protein ECD-Her1 in a batch culture of HEK293 cells.

## Data Availability

The original contributions presented in the study are publicly available. This data can be found here: https://data.mendeley.com/datasets/b83jmwkf3v/1.
