# Supplementary material for: Sodium acetate increases the productivity of HEK293 cells expressing the ECD-Her1 protein in batch cultures: experimental results and metabolic flux analysis
Source: Front Bioeng Biotechnol. 2024 Apr 10;12:1335898. doi: 10.3389/fbioe.2024.1335898 (PMC11039900; doi:10.3389/fbioe.2024.1335898)
Supplement: Supplementary file 1 [file DataSheet1.PDF]

1 SUPPLEMENTARY DATA

1.1 Metabolites and reactions added to the metabolic network of HEK293 cell line

Metabolites:

- **ac_e:** Acetate (extracellular)
- **ac_c:** Acetate (cytosolic)
- **ac_m:** Acetate (mitochondrial)
- **acald_e:** Acetaldehyde (extracellular)
- **acald_c:** Acetaldehyde (cytosolic)
- **acald_m:** Acetaldehyde (mitochondrial)
- **acorn_c:** N2-Acetyl-L-ornithine (cytosolic)
- **Nacasp_c:** N-Acetyl-L-aspartate (cytosolic)
- **amp_m:** AMP C10H12N5O7P (mitochondrial)
- **ppi_m:** Diphosphate (mitochondrial)

Reactions:

1. **EX_ac_e:** Acetate exchange   ac_e $\rightleftharpoons$
2. **ACt2r:** Acetate reversible transport via proton symport   ac_e + h_e $\rightleftharpoons$ ac_c + h_c
3. **ACOAH:** Acetyl-CoA hydrolase   ac_c + coa_c + h_c $\rightleftharpoons$ accoa_c + h2o_c
4. **ACODA:** Acetylornithine deacetylase   acorn_c + h2o_c $\rightleftharpoons$ ac_c + orn_c
5. **NACASPAH:** N-Acetyl-L-aspartate amidohydrolase   h2o_c + Nacasp_c $\rightleftharpoons$ ac_c + asp_L_c
6. **ACt2m:** Acetate mitochondrial transport via proton symport   ac_c + h_c $\rightleftharpoons$ ac_m + h_m
7. **ALDD2x:** Aldehyde dehydrogenase (acetaldehyde, NAD) acald_c + h2o_c + nad_c $\rightleftharpoons$ ac_c + 2.0 h_c + nadh_c
8. **ALDD2y:** Aldehyde dehydrogenase (acetaldehyde, NADP) acald_c + h2o_c + nadp_c $\rightleftharpoons$ ac_c + 2.0 h_c + nadph_c
9. **ACSm**: Acetyl CoA synthetase (mitochondrial) ac_m + atp_m + coa_m $\rightleftharpoons$ accoa_m + amp_m + ppi_m
10. **ACS**: Acetyl CoA synthetase   ac_c + atp_c + coa_c $\rightleftharpoons$ accoa_c + amp_c + ppi_c
11. **CITL:** Citrate lyase   cit_c $\rightleftharpoons$ ac_c + oaa_c

2 ACRONYMS

- **2amac:** 2-Aminoacrylate
- **aaccoa:** Acetoacetyl-CoA
- **ac:** Acetate
- **accoa:** Acetyl-CoA
- **ADP:** Adenosine diphosphate
- **akg:**2-Oxoglutarate
- **ala_L:** L-Alanine
- **arg:** Arginine

- **asn:** Asparagine
- **asp:** Aspartate
- **ATP:** Adenosine triphosphate
- **cit:** Citrate
- **CO_2:** Carbon dioxide
- **coa:** Coenzyme A
- **cys_:** L-cysteine
- **dhap:** Dihydroxyacetone phosphate
- **e4p:** D-Erythrose 4-phosphate
- **f6p:** D-Fructose 6-phosphate
- **fum:** Fumarate
- **g3p:** Glyceraldehyde 3-phosphate
- **g6p:** D-Glucose 6-phosphate
- **glc_D:** D-Glucose
- **glc_D_e:** D-Glucose extracellular
- **gln:** Glutamine
- **glu_L:** L-Glutamate
- **glu5sa:** L-Glutamate 5-semialdehyde
- **gly:** Glycine
- **icit:** Isocitrate
- **ile:** Isoleucine
- **lac_L:** L-Lactate extracellular
- **lac_L:** L-Lactate
- **lys:** Lysine
- **mal_L:** L-Malate
- **met:** Methionine
- **mi1p_D:** 1D-myo-Inositol 1-phosphate
- **mmcoa_R:** (R)-Methylmalonyl-CoA
- **na1:** Sodium
- **sodium acetate:** Sodium acetate
- **NAD:** Nicotinamide adenine dinucleotide
- **NADH:** Nicotinamide adenine dinucleotide - reduced
- **nh3:** Ammonia
- **oaa:** Oxaloacetate
- **pep:** Phosphoenolpyruvate
- **phe:** Phenylalanine
- **pi:** Phosphate
- **pro:** Proline

- **prpp:** 5-Phospho-alpha-D-ribose 1-diphosphate
- **pyr:** Pyruvate
- **r5p:** Alpha-D-Ribose 5-phosphate
- **ru5p_D:** D-Ribulose 5-phosphate
- **s7p:** Sedoheptulose 7-phosphate
- **ser:** Serine
- **succoa:** Succinyl-CoA
- **succ:** Succinate
- **thr:** Threonine
- **val:** Valine
- **xu5p_D:** D-Xylulose 5-phosphate

3 ENERGY CONSUMPTION

Although the ATP yield from the consumption of one mole of acetate in mammalian cells can vary depending on several factors, in general there is a consensus Comerford et al. (2014) that the maximum theoretical yield is approximately 12 mole of ATP per mole of acetate. Moreover, the mean differences of acetate concentration (ΔS_{ac}) in our culture (see Figure S2) was approximately 1.08 mM. Using the equation $u_i = -\mu \frac{ds_i/dt}{dX/dt}$, and the average cell density in the time interval 48-144 hours, where acetate consumption is larger, we can estimate the consumption rate of acetate as:

$$u_{ac} \quad = -\mu \frac{dS_{ac}/dt}{dX/dt} \approx -0.0153 h^{-1} \frac{(-1.08 \text{ mM})}{3.06 \times 10^6 \text{ cell/mL}} \approx 5.4 \times 10^{-15} \text{mol/cell/h.}$$

Considering that one cell of HEK293 type is equivalent to 514×10^{-12} gDW Dietmair et al. (2012), we obtain:

$$\begin{aligned} u_{ac} &\approx \frac{5.4 \times 10^{-15} \text{mol/h}}{514 \times 10^{-12} \text{ gDW}} \\ u_{ac} &\approx 0.0105 \text{ mmol/gDW/h} \end{aligned}$$

Taking account that for each mole of acetate 12 mole of ATP can be produced, the specific ATP production rate is:

$$\begin{aligned} &0.0105 \text{ mmol of Acetate/gDW/h} \times \frac{12 \text{ mmol of ATP}}{1 \text{ mmol of Acetate}} \\ &= 0.126 \text{ mmol of ATP/gDW/h} \qquad \text{ATP production from acetate uptake.} \end{aligned}$$

Now, we estimate the amount of ATP required to produce ECD-Her1 specific protein, in order to be compared with the ATP produced per acetate uptaked.

The average of the experimental protein production rate is:

$$q_p = 0.343 \text{ mg/gDW/h},$$

considering that the molecular weight of ECD-Her1 is 105kDa Duardo et al. (2015), and the conversion ($1\text{kDa} = 10^3$g/mole), we obtain:

$$\begin{aligned} q_p \quad &= 0.343 \times 10^{-3} \text{ g/gDW/h} \times \frac{1\text{mole}}{105 \times 10^3\text{g}} \\ &= 3.27 \times 10^{-6} \text{ mmol of ECD-Her1/gDW/h} \end{aligned}$$

While the number of ATP molecules required per amino acid during protein folding can vary depending on the specific protein and conditions under which it is folded, previous studies Flamholz et al. (2014); Kang et al. (2020) have shown that, on average, approximately 3-5 ATP molecules are required per amino acid (aa) to obtain a proper protein folding. We assume that in our case the protein folding needs 4 ATP molecules per aa. Our protein of interest, ECD-Her1, contains 621 amino acids in its sequence. Therefore, $621\text{aa} \times 4 = 2484$ ATP molecules are required to form the protein.

$$2484 \text{ ATP molecules} \rightarrow 1 \text{ ECD-Her1 molecule}$$

As one mole of a substance contains 6.02×10^{23} molecules of the substance, then

$$2484 \text{ ATP molecules} \frac{1 \text{ mole}}{6.02 \times 10^{23} \text{ molecules}} \rightarrow 1 \text{ ECD-Her1 molecule} \frac{1 \text{ mole}}{6.02 \times 10^{23} \text{ molecules}}$$

And we obtain,

$$2484 \text{ ATP mole} \rightarrow 1 \text{ ECD-Her1 mole}$$

With this, we can estimate the ATP requirement to produce the ECD-Her1 specific protein

$$\begin{aligned} &3.27 \times 10^{-6} \text{ mmol of ECD-Her1/gDW/h} \times \frac{2484 \text{ mmol of ATP}}{1 \text{ mmol of ECD-Her1}} \\ &= 8.94 \times 10^{-3} \text{ mmol of ATP/gDW/h} \qquad \text{ATP requirement per mole of ECD-Her1} \end{aligned}$$

$$\frac{\text{ATP production from acetate uptake}}{\text{ATP requirement for protein folding}} \approx 14 \text{ fold}$$

4 FIGURES

REFERENCES

Comerford, S. A., Huang, Z., Du, X., Wang, Y., Cai, L., Witkiewicz, A. K., et al. (2014). Acetate dependence of tumors. *Cell* 159, 1591–1602

Dietmair, S., Hodson, M. P., Quek, L.-E., Timmins, N. E., Gray, P., and Nielsen, L. K. (2012). A multi-omics analysis of recombinant protein production in HEK293 cells. *PLoS One*

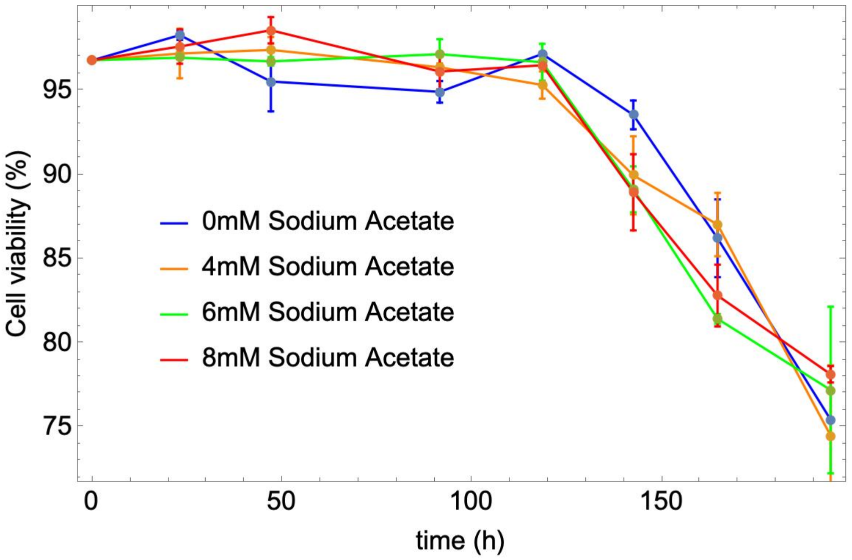

Figure S1. Cell viability of HEK293 cell culture at differents concentrantions of sodium acetate (0 mM, 4 mM, 6 mM and 8 mM).

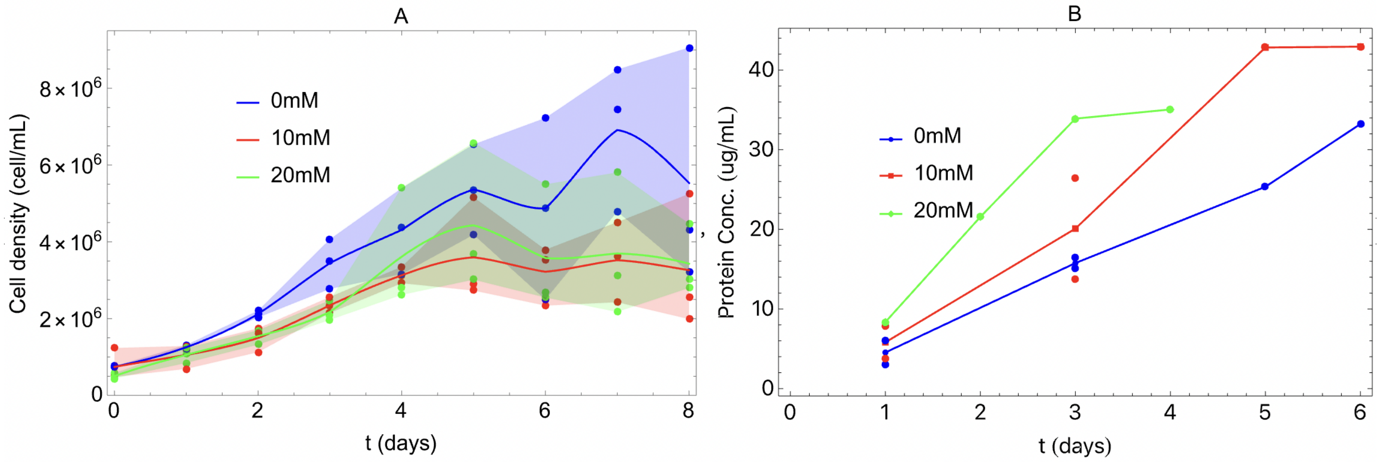

Figure S2. Cell density (A) and protein concentrations (B) of HEK293 cell culture at differents concentrantions of sodium acetate (0 mM, 10 mM, and 20 mM).

Duardo, K. G., Curbelo, Y. P., Pous, J. R., Legón, E. Y. R., Ramírez, B. S., de la Luz Hernández, K. R., et al. (2015). Assessment of the impact of manufacturing changes on the physicochemical properties and biological activity of Her1-ECD vaccine during product development. *Vaccine* 33, 4292–4299

Flamholz, A., Phillips, R., and Milo, R. (2014). The quantified cell. *Molecular Biology of the Cell* 25, 3497–3500

Kang, J., Lim, L., and Song, J. (2020). Atp induces protein folding, inhibits aggregation and antagonizes destabilization by effectively mediating water-protein-ion interactions, the heart of protein folding and aggregation. *BioRxiv* , 2020–06

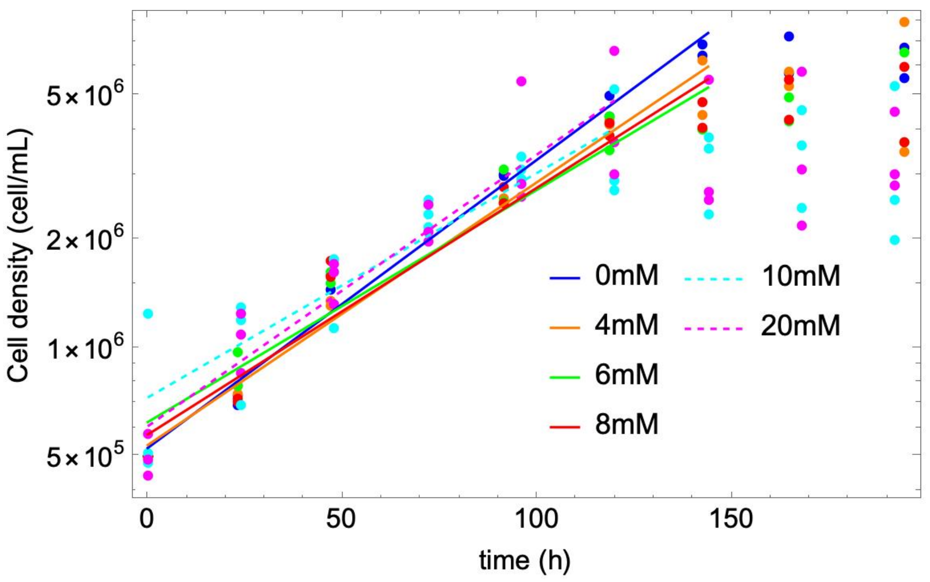

Figure S3. Cell density in logarithmic scale versus time at different initial concentrations of sodium acetate (0mM, 4 mM, 6mM, 8mM, 10mM and 20mM). Each line is an interpolation using the mean of cell density measured.

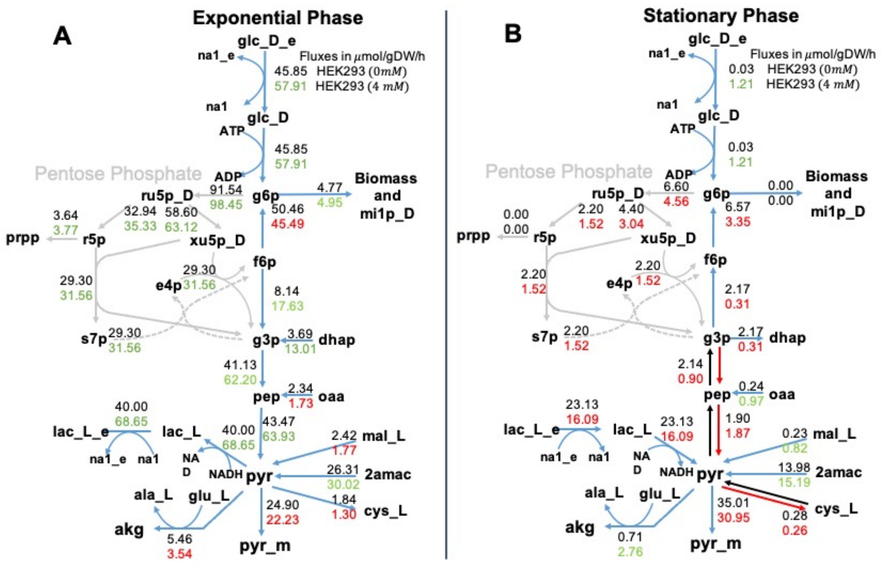

Figure S4. Metabolic fluxes in glycolysis. There are two maps, on the left, the exponential phase (panel A), and on the right the stationary phase (panel B). We represent two values for each metabolic reaction shown, the top value is the flux of the control (0 mM of sodium acetate) and the bottom value is the flux at 4 mM of sodium acetate. We used red to represent fluxes that were lower than the control value, green to represent fluxes that were higher, and black to represent fluxes that were equal. The subscript _e corresponds to extracellular metabolites, _m to mitochondrial metabolites, and the rest of the metabolites are found in the cytosol.

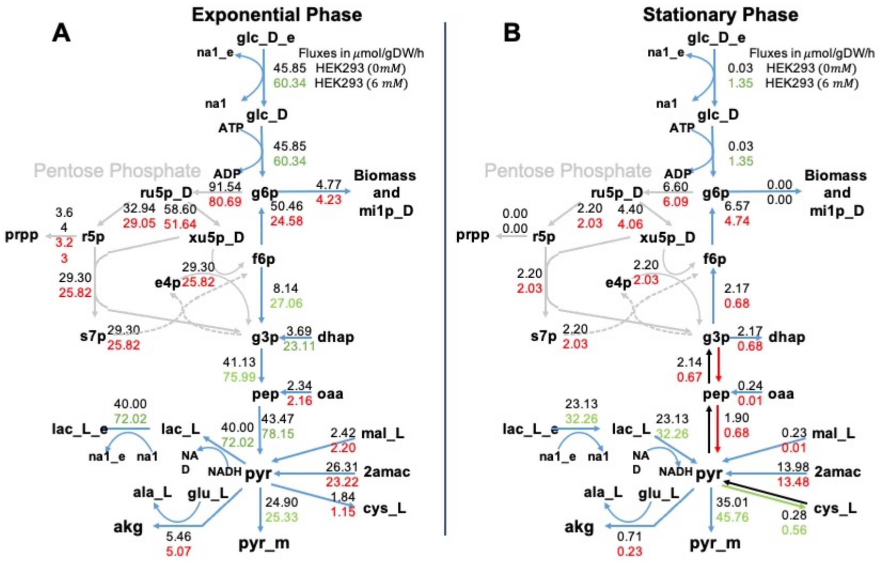

Figure S5. Metabolic fluxes in glycolysis. There are two maps, on the left, the exponential phase (panel A), and on the right the stationary phase (panel B). We represent two values for each metabolic reaction shown, the top value is the flux of the control (0 mM of sodium acetate) and the bottom value is the flux at 6 mM of sodium acetate. We used red to represent fluxes that were lower than the control value, green to represent fluxes that were higher, and black to represent fluxes that were equal. The subscript _e corresponds to extracellular metabolites, _m to mitochondrial metabolites, and the rest of the metabolites are found in the cytosol.

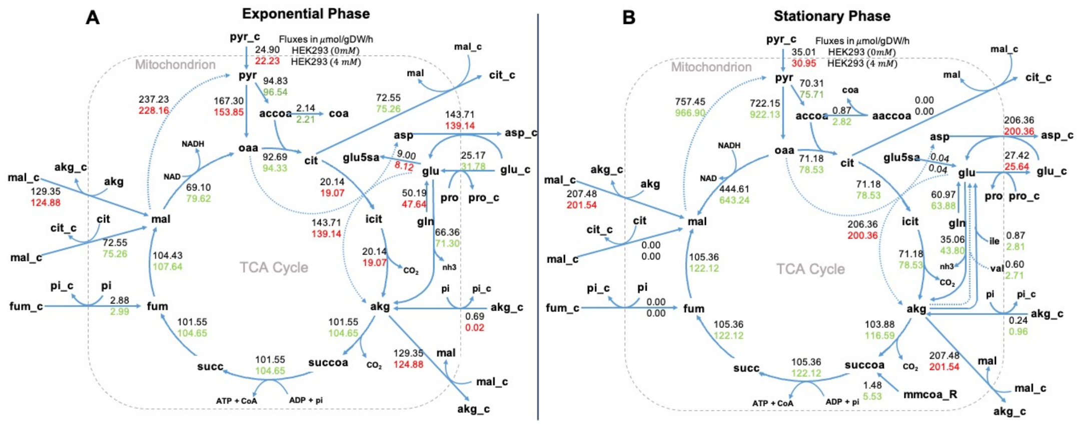

Figure S6. Metabolic fluxes in TCA cycle. There are two maps, at the top, exponential phase (panel A), and at the bottom stationary phase (panel B). We represent two values for each metabolic reaction shown, the top value is the flux of the control (0 mM of sodium acetate) and the bottom value is the flux at 4 mM of sodium acetate . We used red to represent fluxes that were lower than the control value, green to represent fluxes that were higher, and black to represent fluxes that were equal. _e corresponds to extracellular metabolites, _c to cytosolic and _m to mitochondrial.

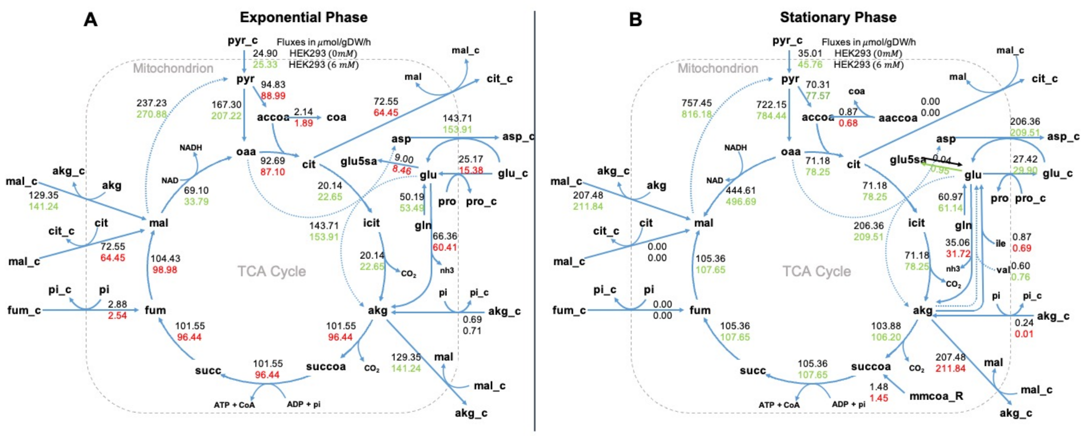

Figure S7. Metabolic fluxes in TCA cycle. There are two maps, at the top, exponential phase (panel A), and at the bottom stationary phase (panel B). We represent two values for each metabolic reaction shown, the top value is the flux of the control (0 mM of sodium acetate) and the bottom value is the flux at 6 mM of sodium acetate . We used red to represent fluxes that were lower than the control value, green to represent fluxes that were higher, and black to represent fluxes that were equal. _e corresponds to extracellular metabolites, _c to cytosolic and _m to mitochondrial.
